# The Parkinson Disease gene SNCA: Evolutionary and structural insights with pathological implication

**DOI:** 10.1038/srep24475

**Published:** 2016-04-15

**Authors:** Irum Javaid Siddiqui, Nashaiman Pervaiz, Amir Ali Abbasi

**Affiliations:** 1 National Center for Bioinformatics, Program of Comparative and Evolutionary Genomics, Faculty of Biological Sciences, Quaid-i-Azam University, Islamabad 45320, Pakistan

## Abstract

After Alzheimer, Parkinson’s disease (PD) is the second most common neurodegenerative disorder. Alpha synuclein (SNCA) is deemed as a major component of Lewy bodies, a neuropathological feature of PD. Five point mutations in SNCA have been reported so far, responsible for autosomal dominant PD. This study aims to decipher evolutionary and structural insights of SNCA by revealing its sequence and structural evolutionary patterns among sarcopterygians and its paralogous counterparts (SNCB and SNCG). Rate analysis detected strong purifying selection on entire synuclein family. Structural dynamics divulges that during the course of sarcopterygian evolutionary history, the region encompassed 32 to 58 of N-terminal domain of SNCA has acquired its critical functional significance through the epistatic influence of the lineage specific substitutions. In sum, these findings provide an evidence that the region from 32 to 58 of N-terminal lipid binding alpha helix domain of SNCA is the most critical region, not only from the evolutionary perspective but also for the stability and the proper conformation of the protein as well as crucial for the disease pathogenesis, harboring critical interaction sites.

PD is the second most common neurodegenerative disorder after Alzheimer which affects 1–2% of the population above age 65 and 4–5% above age 85^1^. It is characterized by the loss of dopaminergic neurons from substantia nigra (a brain structure located in the mesencephalon that plays an important role in reward, addiction, and movement) and the presence of intracellular inclusions i.e. lewy bodies and lewy neuritis^2^. Symptoms of PD include shaking, tremors, bradykinesia and difficulty with walking and gait. Other symptoms include sensory, sleep and emotional problems. SNCA is considered as the major causative gene involved in the early onset of familial Parkinson’s disease (FPD) characterized by five missense mutations identified so far i.e. A30P^3^, E46K^4^, H50Q^5^, G51D^6^ and A53T^7^. SNCA is also deemed to be involved in various other neurodegenerative disorders i.e. Alzheimer’s disease (AD), Lewy bodies’ disease (LBD) and Muscular System Atrophy (MSA)^8^.

SNCA is a 14.5 kDa, 140 a.a protein encoded by 5 exons with total transcript length of 3041 bps maps on 4q21.3-q22. The other members of synuclein family are SNCB and SNCG mapped to human chromosome 5q35 and 10q23.2-q23.3 respectively^9^. Architecture of SNCA protein reveals the presence of N-terminal region composed of incomplete KXKEGV motifs, extremely hydrophobic NAC domain and highly acidic C-terminal domain^8^. At physiological conditions, SNCA is believed to be intrinsically disordered monomer or helically folded tetramer^10^. Oligomeric structure of SNCA is considered as a toxic form but recent observation abolished this hypothesis^10^,^11^. During the past two decades several hypotheses exist about toxic structural form of SNCA, but none of them are completely consensual. However, neurotoxic form of SNCA aggregates within neuron and spreads across the anatomically interconnected regions of PD brain through interneuronal transmission using various mechanisms^12^. Although SNCA is expressed predominately in brain, it is also expressed in heart, skeletal muscle and pancreas^8^,^13^. Molecular function of SNCA is quite ambiguous. Based on its structure, physical properties and interacting partners, several hypotheses for the normal function of SNCA have been proposed. It is considered to be involved in regulation of dopamine release and transport, induces fibrillization of microtubule associated protein tau, and exert neuroprotective phenotype in non-dopaminergic neurons by inhibiting both p53 expression and transactivation of proapoptotic genes leading to decreased caspase-3 activation^8^,^13^,^14^,^15^,^16^. However, missense mutations (especially A53T) in SNCA abolished the neuroprotective effect of SNCA and promote apoptosis by reversing the expression of p53^14^,^15^.

Due to the significant role of SNCA in FPD and other neurodegenerative disorders, this study was premeditated to decipher the molecular evolution of SNCA, which infers the phylogenetic history of synuclein family with the help of its putative orthologs and paralogs. Analysis revealed the sarcopterygian specific origin of SNCA which suggested its lineage specific functional role. On account of this interest, a comparative sequence and structural analysis was performed to estimate the selection and functional constraints on SNCA. Evolutionary rate difference was coupled with structural information to infer potential functional changes and the impact of lineage specific substitutions. In addition variations in domain topologies were explored by comparative analysis of known functional domains of SNCA protein. In light of the findings, it was hypothesized that the region from 32 to 58 of N-terminal lipid binding domain is the most *“critical region”* of SNCA from evolutionary, functional and disease pathogenesis perspective.

## Materials and Methods

### Sequence collection

Putative paralogs of human SNCA were determined by Ensembl Genome Browser^17^ using Ensembl paralogy prediction. The closest putative orthologs were obtained by BLASTp^18^ bidirectional searches against protein database available at Ensembl and National Centre for Biotechnology Information (NCBI). Confirmation about ancestral-descendents relationship among putative orthologs was done through clustering of homologous proteins within phylogenetic trees. Sequences whose position within a tree was sharply in conflict with the uncontested animal phylogeny were excluded. The list of all used sequences (protein sequence data) is given as Supplementary Data File 1.

Species that were selected includes *Homo sapiens* (Human), *Pan troglodytes* (Chimpanzee), Mus musculus (Mouse), *Rattus norvegicus* (Rat), *Gallus gallus* (Chicken), *Canis familiaris* (Dog), *Equus caballus* (Horse), *Loxodonta Africana* (Elephant), *Dasypus novemcinctus* (Armadillo), *Anolis carolinensis* (Lizard), *Pelodiscus sinensis* (Chinese softshell turtle), *Xenopustropicalis* (Frog), *Latimeria chalumnae* (Coelacanth), *Danio rerio* (Zebrafish), *Takifugu rubripes* (Fugu), *Tetraodon nigroviridis* (Tetraodon), *Gasterosteus aculeatus* (Stickleback), *Oryzias latipes* (Medaka).

### Sequence analysis

The phylogenetic tree of SNCA family was reconstructed by using the neighbor-joining (NJ) method^19^,^20^, complete deletion option was used to exclude any site which postulated a gap in the sequences. Poisson corrected (PC) amino acid distance and uncorrected proportion (p) of amino acid difference were used as amino acid substitution models. Because both methods produced similar results, only results from NJ tree based on uncorrected p-distance are presented here. Reliability of the resulting tree topology was tested by the bootstrap method^21^ (at1000 pseudo replicates) which generated the bootstrap probability for each interior branch in the tree. Maximum Likelihood (ML) tree was also constructed by using the Whelan and Goldman^22^ (WAG) model of amino acid replacement (see Supplementary Fig. S1).

Ancestral SNCA sequences were inferred by using ML method and WAG model of amino acid substitution. To investigate selection constraint within hominoids (human, chimpanzee, gorilla, orangutan), non-hominoids (macaque, marmoset, squirrel monkey, bushbaby), non-primate placental mammals (mouse, dog, cow, elephant) and non-mammalian tetrapods (chicken, turtle, frog, coelacanth), z-test was implemented with MEGA^23^. dN-dS rate analysis was also conducted for each of the above mentioned group with the help of Goldman And Yang^24^ (GY-94) method in Hyphy which estimates synonymous and non-synonymous substitution rates through codon based model^24^. Evolutionary rate differences of putative paralogs (SNCB & SNCG) were also assessed among sarcopterygians.

Domains, motifs and sub-motifs have been assigned to human SNCA as described previously^25^,^26^. ClustalW2 based multiple sequence alignments were used to map the putative positioning of these domains and motifs to paralogs of SNCA protein in human and its orthologs in various sarcopterygian species^27^. Location and the positioning of the identified domains and motifs were also plotted. Substitutions that have occurred within sarcopterygians during evolution were assigned to human SNCA with the help of ancestor reconstruction technique. Previously reported human specific mutations involved in familial Parkinson’s disease were also mapped. Negatively constrained residues of SNCA among sarcopterygians were estimated with Hyphy by implementing Single Likelihood Ancestor Counting (SLAC) method which uses global codon model and maximum likelihood to reconstruct the evolutionary history^24^. Impact of the substitutions that have occurred during evolution within sarcopterygians with K_a_/K_s_ <1 were also classified on the basis of their physicochemical properties i.e. charge, volume, polarity into neutral or radical^28^,^29^. Human paralogs of SNCA (SNCB & SNCG) were likewise compared and paralogs specific substitutions were identified.

### Structural analysis

For the structural analysis with evolutionary and functional perspective, NMR structure of human SNCA (1XQ8) was attained from Protein Data Bank (PDB)^30^ and used as a reference for comparative analysis of the structural deviations. After inferring ancestral sequences, sarcopterygian ancestral, mammalian and non-primate placental mammal’s specific SNCA proteins were modeled with the help of Modeller^31^. Structures of the putative paralogs (SNCB & SNCG) of human SNCA were also modelled by comparative homology modelling. Best structures were scrutinized on the basis of Discrete Optimized Protein Energy (DOPE) score, and then further energy minimization protocol was implemented with chimera^32^ in order to improve the quality of the modeled structures. Quality of the modeled structures were also investigated by Ramachandran^33^ and Errat^34^ plots (see Supplementary Fig. S2A,B, S5A,B). Superimposition of the modeled structures with 1XQ8 was carried out with chimera^32^ and root mean square deviation (RMSD) values were calculated. Quantification of the structural deviations observed in chimera^32^ were further reconnoitered by SPINEX^35^ in terms of quantification of the deviations identified in the backbone torsion angles (phi Φ°, psi Ψ°) of the modeled ancestral proteins. Impact of the lineage specific substitutions on the modeled ancestral SNCA proteins were investigated with the help of MuPro^36^ in terms of sequence and structural stability.In order to inspect the structural deviations in the human specific mutations i.e. A30P, E46K, H50Q, G51D, A53T involved in FPD, mutant models were also generated by Modeller^31^, analyzed and minimized by chimera^32^ and estimated with Ramachandran^33^ and Errat^34^ (see Supplementary Figs S3 and S4A,B).

### Interaction study

To conduct the interaction study, Cluspro protein-protein docking server^37^ was utilized. NMR structure of coiled-coil domain (2KES) of the interacting partner, synphilin-1 (SNCAIP) was obtained from PDB^38^. Domains and motifs reported in literature were assigned to human SNCAIP. Interactions between human specific (1XQ8), sarcopterygian ancestral, mammalian, non-primate placental mammal’s specific and human specific mutant models of SNCA proteins were examined with the help of Ligplot^39^ and PyMol^40^.

## Results

### Phylogenetic analysis

**Figure 1:**
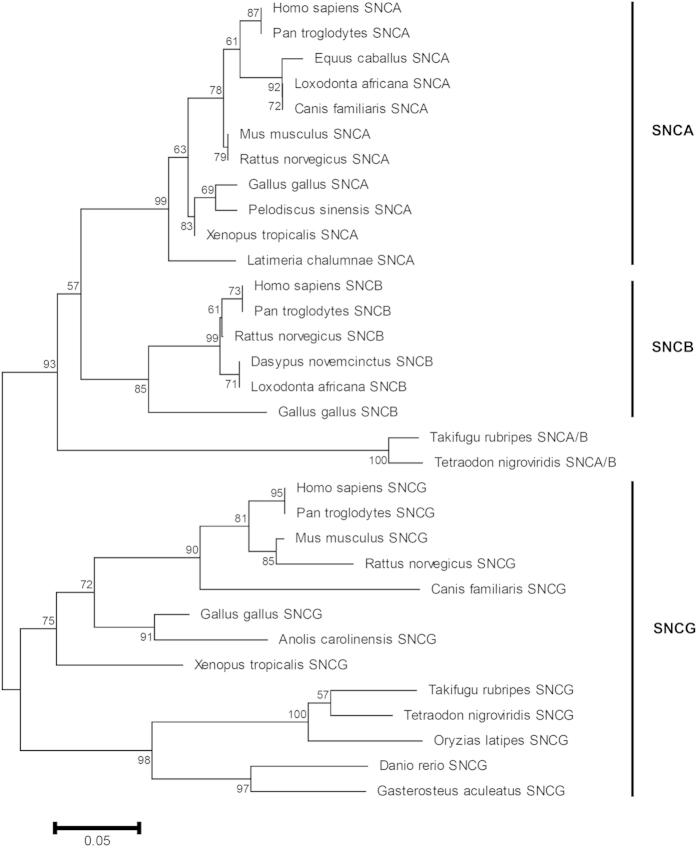
Neighbor Joining tree of the synuclein family members. Uncorrected p-distance was used. Complete-deletion option was used. Numbers on branches represent bootstrap values (based on 1000 replications) supporting that branch; only the values ≥50% are presented here. Scale bar shows amino acid substitution per site.

Evolutionary relationship between SNCA and its putative paralogs was estimated by NJ and ML methods (Fig. 1, see Supplementary Fig. S1). Phylogenetic analysis of synuclein family revealed that two duplication events have contributed in diversification of this family. The first duplication event has transpired at the root of vertebrates, prior to tetrapod-teleost split, deduced in SNCG paralog and ancestor of SNCA/SNCB. Whereas the second duplication event has occurred at the root of sarcopterygian’s lineage, after speciation of the teleost fish (SNCA/B) resulted in SNCA and SNCB paralogs. It is also worth noting that the branch lengths for SNCG proteins are longer than those of other two paralogs, suggesting that this paralog may have rapidly evolved in comparison with SNCA and SNCB. Blast based bidirectional similarity searches fail to identify any ortholog of this family among invertebrates which reinforces the assumption of vertebrate specific origin of this family (Fig. 1, see Supplementary Fig. S1).

### Comparing evolutionary rate of SNCA gene among sarcopterygians

In order to estimate the evolutionary rate differences of SNCA gene among various clades of sarcopterygians, the orthologs of SNCA from representative members of hominoids (human, chimpanzee, gorilla, orangutan), non-hominoids (macaque, marmoset, squirrel monkey, bushbaby), non-primate placental mammals (mouse, dog, cow, elephant) and non-mammalian tetrapods (chicken, turtle, frog, coelacanth) were obtained. Non synonymous (K_a_/dN) and synonymous substitution rates (K_s_/dS) were estimated for each group. And then z-test was applied to check selection constraint on the above mentioned groups.

The K_a_-K_s_ (dN-dS) difference for hominoids was found as −2.241 (P = 0.014), non-hominoids was −4.716 (P = 0), non-primate placental mammals was −6.1777 (P = 0) and non-mammalian tetrapods was −7.085 (P = 0) (see Supplementary Table S1). In general, K_a_ value lower than K_s_ (K_a_ < K_s_) suggests negative selection, i.e. non silent substitutions have been purged by natural selection, whereas the inverse scenario (K_a_ > K_s_) implies positive selection i.e. advantageous mutations have accumulated during the course of evolution. However the evidence for positive or negative selection requires the value to be significantly different from each other^41^,^42^. Results deduced by z-test (Ka-Ks < 0, p < 0.05) suggests that SNCA has deviated from neutrality during evolution showing the signature of negative selection constraint within sarcopterygian lineage (see Supplementary Table S1).

The evolutionary rate analysis were also performed for putative paralogous copies of SNCA, i.e. SNCB and SNCG. The K_a_-K_s_ (dN-dS) difference for SNCB was identified as −1.661(P = 0.05) for hominoids, −4.708(P = 0) for non-hominoids primates, −5.212(P = 0) for non-primate placental mammals and −2.992(P = 0.002) for non-mammalian tetrapods (see Supplementary Table S2). The K_a_-K_s_ (dN-dS) difference for SNCG was identified as −1.658(P = 0.05) for hominoids, −4.064(P = 0) for non-hominoid primates, −5.485(P = 0) for non-primate placental mammals, −6.306(P = 0) non-mammalian tetrapods and −6.341(P = 0) for fishes (fugu, tetraodon, stickleback, medaka) (see Supplementary Table S3). This data specifies that not only SNCA but other two members of synuclein family have also been retained under strong purifying selection pressure among the analyzed sarcopterygians.

### Domain organization of SNCA

**Figure 2:**
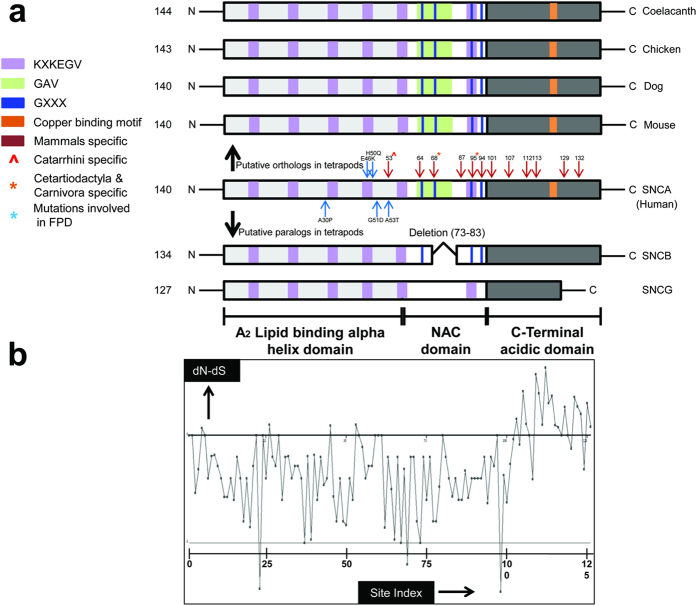
Schematic representation. (**a**) Domain organization of SNCA protein. Schematic view of comparative organization of key functional domains and motifs of SNCA across human paralogous and orthologous proteins from phylogenetically distant species. Protein lengths are drawn approximately to scale. Domains and motifs are color coded. (**b**) Window displaying the sites under the negative selection constraint in SNCA among sarcopterygians. Results are generated with Hyphy by implementing SLAC method which uses global codon model and maximum likelihood to reconstruct the evolutionary history.

In order to gain an insight into comparative domain organization, complete domain annotation of SNCA gene was carried out entailing the orthologs representative from sarcopterygians (human, mouse, dog, chicken, coelacanth) as well as the paralogous copies in human (SNCB and SNCG). This annotation revealed the distinctive architecture of SNCA gene which is comprised of N-terminal A_2_ lipid binding alpha helix domain (1–60), Non-amyloid β component (NAC) domain (61–95) and C-terminal acidic domain (96–140) (Fig. 2a).

N-terminal lipid binding domain consists of 5 KXKEGV imperfect repeats^26^, and these repeats are identified as highly conserved among analyzed orthologs and paralogs of human SNCA in terms of number and position (Fig. 2a). This region is predicted to form amphipathic α-helices, and considered to be involved in interacting with phospholipids^26^.

NAC domain forms the amyloidogenic core of SNCA^26^. NAC comprised of GAV motif with VGGAVVTGV(66–74) consensus sequence and three GXXX sub-motifs (where X is any of Gly, Ala,Val, Ile, Leu, Phe, Tyr, Trp, Thr, Ser or Met)^26^. Among the analyzed orthologs, NAC was identified as highly conserved. Whereas, among its counter paralogous copies, in SNCB the length of NAC (61–84) was reduced due to the absence of one GXXX motif and KXKEGV repeat. While no GXXX sub-motif was identified in SNCG. Among the three putative paralogs, the GAV motif was explicitly present in SNCA only (Fig. 2a). NAC is considered as extremely necessary for SNCA aggregation and fibrillation^26^. Whereas GAV is surmised as signature motif responsible for this aggregation process^25^.

C-terminal acidic domain harbors copper binding motif containing DPDNEA(119–124) consensus sequence^43^ which was found highly conserved among the analyzed orthologs of SNCA. Multiple sequence alignment failed to identify the conservation of this motif among SNCB and SNCG paralogs (Fig. 2a). This domain of SNCA is enriched in acidic residues and prolines. Three highly conserved tyrosine residues, which are considered as a subfamily signature of SNCA and SNCB, are also located in this region^26^. It is also proposed that copper binding accelerates the aggregation of SNCA and influences its pathological effects^44^.

Lineage specific substitutions that have occurred during evolution among analyzed sarcopterygians have been mapped on human SNCA with the help of ancestor reconstruction technique. It classified that nine substitutions have occurred at the root of mammalian ancestor. While two substitutions occurred at the root of non-primate placental mammal’s lineage and one occurred specifically to the catarrhini’s (hominoids and old world monkeys) lineage (Fig. 2a) (Table 1). Out of these 12 substitutions identified, five (S64T, G68E, N87S, L94F, V95G) were found to be confined towards NAC, while six (A101G, F107A, M112I, M113L, P129S, E132G) were residing in the C-terminal acidic domain. Only 1 substitution (T53A) was identified in the N-terminal region (Fig. 2a). Physicochemical properties of these amino acid substitutions were then analyzed which illustrated that approximately all the substitutions that have occurred during evolution were of radical type except T53A and A101G (Table 1). This analysis highlights N-terminal lipid binding domain as highly conserved among the analyzed orthologs and paralogs. This finding was further strengthened by the physical positioning of previously reported five human specific mutations associated with familial Parkinson’s disease i.e. A30P^3^, E46K^4^, H50Q^5^, G51D^6^, and A53T^7^ on human SNCA. Results revealed the confinement of these mutations explicitly towards the N-terminal domain which in turn implies the significance of high conservation of this region not only with functional perspective but also for pathogenesis of FPD (Fig. 2a). With the help of SLAC-window analysis, it appears that N-terminal domain comprised of 15 negatively constrained sites which further advocates that strong selective constraints are operating their role in preserving this region during sarcopterygians evolution (Fig. 2b, see Supplementary Table S4).

### Structural evolution of SNCA

**Figure 3:**
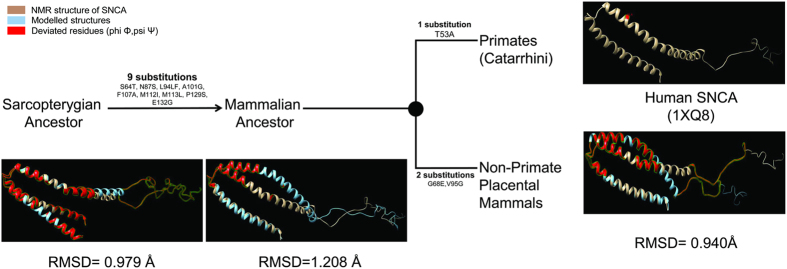
Structural evolution of SNCA proteins since the split from last common sarcopterygian ancestor. Significant structural divergence towards human SNCA was observed after the split of common sarcopterygian ancestor. Nine specific substitutions have occurred at the root of mammalian lineage which is retained in primates and the non-primate placental mammals after the split from sarcopterygian ancestor. Whereas, two substitutions have occurred specifically at the root of non-primate placental mammals lineage and one catarrhini specific substitution has occurred after the split from common mammalian ancestor. Deviated residues in terms of backbone torsion angles (Φ°,Ψ°) from the human SNCA (1XQ8) are represented in red color. Structural deviations were examined by RMSD values.

To further inspect how purifying selection is performing its role in defining the spatial constraints on ancestral SNCA proteins at structural level, a comparative structural study was conducted. NMR structure of human SNCA (1XQ8) was taken as a reference and compared with modeled ancestral proteins (Fig. 3). Structural deviations were examined with the help of RMSD values (Fig. 3, see Supplementary Fig. S2A,B). Results revealed very remarkable aspects that were not anticipated by comparative analysis at sequence level. Comparative structural analysis suggests that structure of SNCA has passed through series of transitions to acquire its favored conformation. Superimposed models of ancestral SNCA proteins and 1XQ8 revealed common deviated region encompassed 32 to 58 of N-terminal lipid binding domain of SNCA (Table 2). These structural deviations were also measured with the help of backbone torsions quantification which highlighted the fact that the region 32 to 58 of SNCA is continuously evolving at structural level, despite of its high sequence conservation (Table 2). It was also identified that destabilizing substitutions have been incorporated during SNCA evolution with the aim to achieve its intrinsic disordered conformation which implies the functional constraints behind it (Table 1). So it seems logical to speculate from this structural comparison approach that during SNCA evolution those substitutions have been incorporated which not only caused destabilization of SNCA but also brought drastic structural shift in the identified region. This critical region (32–58) was also recognized crucial for the proper conformation of not only N-terminal A_2_ alpha helix domain but for NAC domain as well. With the help of electron and X-ray diffraction techniques it has been reported that normal SNCA assembles through its N-terminal region which again highlights the significant role of this domain^45^.

Intriguingly, all human specific mutations involved in FPD pathogenesis reside in this crucial region which signifies that any change in this region will be deleterious because of the strong selection and functional constraints imposed on it. Superimposed mutant models with 1XQ8 identified major shifts toward lipid binding domain in A30P and H50Q, whereas major change was observed in lipid binding and NAC domains in case of E46K and A53T. Only G51D showed altered NAC region only (see Supplementary Fig.S4A,B). All five mutant models were having highly deviated region from 32 to 58 in common. It can be postulated from this comparative structural analysis that the primary effects and the role of these five SNCA mutations in FPD pathogenesis can be different because of their differential structural morphologies.

**Figure 4:**
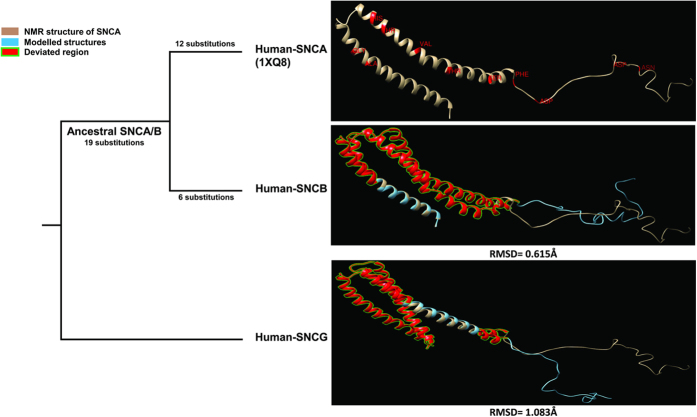
Structural divergence among human paralogs of SNCA. Major structural shifts were observed in N-terminal lipid binding and NAC domains due to paralogous specific substitutions. After 1^st^ duplication event ancestral SNCA/B experienced 19 changes. After 2^nd^ duplication SNCB and SNCA experienced 6 and 12 substitutions respectively. Deviated residues in comparison with human SNCA (1XQ8) are color coded. Structural deviations were assessed by RMSD values.

Further to investigate the structural differences among the human paralogs of SNCA, a comparative structural analysis was also conducted. As the NMR structures of human SNCB and SNCG have not been reported so far, their structures were modelled by taking NMR structure of human SNCA (1XQ8) as reference and the structural deviations were assessed (see Supplementary Fig.S5A,B). It appears that SNCB and SNCG structures are highly deviated from SNCA at N-terminal and NAC domain (Fig. 4).

### Analysis of the interactions between SNCA and coiled-coil domain of SNCAIP

**Figure 5:**
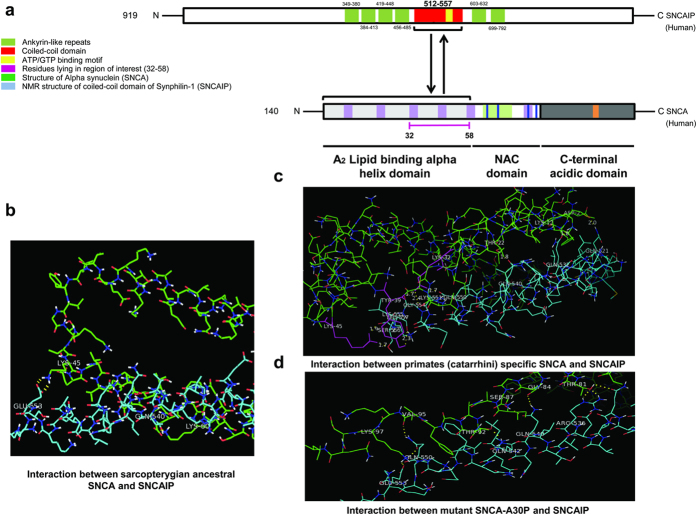
Schematic view (**a**) Domain organization of SNCA and SNCAIP proteins. Comparative organization of key functional domains and motifs of human SNCA and coiled-coil domain of human SNCAIP. Protein lengths are drawn approximately to scale. Domains and motifs are color coded. (**b**) Analysis of the docked complexes and hydrogen bond interactions. Display interactions between the sarcopterygian ancestral SNCA and coiled-coil domain of SNCAIP (2KES). (**c**) Represent interactions between the human SNCA (1XQ8) and coiled-coil domain of human SNCAIP. Interacting residues lying in the region of interest (32–58) are color coded. (**d**) Shows interaction between the modeled mutant of SNCA-A30P and the coiled-coil domain of human SNCAIP. Dotted lines are showing hydrogen bonding.

In order to investigate further the importance of this critical region, its functional significance was then deciphered with the help of interaction study. For this purpose, synphilin-1 (SNCAIP) was considered, as domain annotation of synphilin-1 revealed that it is 919 a.a (3745 bp) protein encoded by 10 exons^17^, encompassed six ankyrin like repeats and one central coiled-coil domain (510–557) (Fig. 5a). It has been corroborated from biochemical and NMR techniques that SNCAIP interacts with N-terminal region of SNCA^38^. Although the normal cellular function of these interacting partners is still unknown but it has been reported that SNCAIP is developmentally localized to synaptic terminals and its association with synaptic vesicles is modulated by SNCA. In this context, SNCAIP is regarded as synaptic partner of SNCA, implying that this interaction mediates the synaptic functions of SNCA, possibly by anchoring SNCA to the vesicle membrane^46^.

In order to explore the role of critical region in interaction, docking analysis was conducted. Interactions have been identified between sarcopterygian ancestral, mammalian specific and non- primate placental mammal’s specific SNCA proteins, which revealed that interaction between SNCA and coiled-coil domain of SNCAIP has evolved with the passage of time i.e. lineage specific interactions have emerged during sarcopterygian evolutionary history (Fig. 5b). Interaction analysis between human specific SNCA and coiled-coil domain of SNCAIP revealed that at the root of catarrhines, few lineage specific interactions have evolved i.e. Lys32, Tyr39, and Lys45 (see Supplementary Fig. S6, see Supplementary Table S5). Interestingly these human (catarrhines) specific interactions reside in the identified critical region which strengthens our hypothesis of the structural and functional significance of this region and its vital role in FPD pathogenesis (Fig. 5c).

Interaction analysis between mutant models of human specific SNCA with SNCAIP revealed altered interaction patterns while some of the wild type interactions were retained too which signifies that SNCA and coiled-coil domain of SNCAIP not only interact in normal individuals but also in the FPD patients, however the pattern of interactions was found altered. Docked complexes of A30P-SNCAIP and E46K-SNCAIP revealed that the interactions shifted entirely to the NAC domain whereas H50Q-SNCAIP and G51D-SNCAIP complexes showed altered interactions involving N-terminal and NAC domains. Only interactions in A53T-SNCAIP were confined entirely to N-terminal domain but the pattern was found altered. It can be assumed that the interactions altered due to differential interaction pattern of SNCA and SNCAIP thus affecting their binding affinities which in turn influence SNCA aggregation (Fig. 5d, see Supplementary Fig.S7, see Supplementary Table S6).

## Discussion

Advent of high throughput annotation of genes has enabled bioinformatics analysis of genes of interest to provide important insight into their evolutionary link with particular phenotypic trait and association with human disease^41^. SNCA is deemed as one of the major causative genes in different neurodegenerative disorders. Five point mutations in SNCA have been reported so far, responsible for autosomal dominant Parkinson’s disease. This novel study primarily highlights the sequence and structural mechanisms of SNCA protein evolution within sarcopterygian lineage, in particular for the first time, with the precise role of the lineage specific substitutions.

The ML and NJ gene phylogenies of synuclein family revealed that SNCA is a sarcopterygian specific gene and thus pinpoints its functional specificity to this class. The three putative paralogs of this family form the (SNCA,SNCB)(SNCG) topology indicating that SNCA and SNCB are closely related duplicate genes. Both of these paralogs share 63% identity which is also depicted in their cellular localization and function^17^ (Fig. 1, see Supplementary Fig. S1). For instance, SNCA and SNCB are expressed predominately in brain, particularly enriched at presynaptic terminals performing membrane associated processes^9^. SNCG share 53% sequence identity with SNCA^17^. The divergent phylogenetic positioning of SNCG might account for differences in the functional aspects of this protein and its putative paralogous counter parts in vertebrates^9^. Blast searches complemented by phylogenetic data confirm the absence of ortholog of this family in invertebrates.

Branching pattern of the phylogenetic tree of synuclein family revealed that the two putative paralogs, SNCA and SNCB are evolving relatively at a slower rate as compared to SNCG. This finding led us to examine the molecular evolution of SNCA and its putative paralogous genes (SNCB and SNCG) specifically in sarcopterygian lineage. For this purpose the average K_a_ (dN) and K_s_ (dS) values were calculated within different phylogenetic groups of sarcopterygians. Estimation of statistical significance of difference between average K_a_ and K_s_ (z-test) within each phylogenetic groups of sarcopterygians have shown signature of strong negative selection on SNCA and its putative paralogous copies (SNCB & SNCG) (see Supplementary Table S1, S2, S3) which corresponds to the structural and functional constrains on synuclein family. Particularly, the selective constraints on SNCA (being a major causative player in FPD) during sarcopterygians evolution is also depicted in highly conserved domain organization among its orthologous and paralogous proteins. Surveying domain topologies revealed highly preserved domain features: N-terminal A_2_ alpha helix lipid binding domain, hydrophobic NAC domain and an acidic C-terminal domain. Explicitly, the N-terminus was identified as highly preserved among the analyzed orthologs and paralogs. This finding has directed this study to inspect the role of this region in defining the spatial constraints during the structural evolution of SNCA protein (Figs 2a,b).

Comparative structural analysis of human SNCA with its ancestral and mutant proteins and also with its paralogous proteins in human (SNCB and SNCG) revealed significant structural deviations encompassing region 32 to 58 of N-terminal lipid binding domain of SNCA, despite of its high sequence conservation (Figs 3 and 4, Table 2, see Supplementary Figs S2A,B, S4A,B and S5A,B). The destabilization of SNCA observed through structural evolution can be best explained by speculating that such intrinsic disordered proteins undergo transitions to more ordered states upon binding to their targets (Table 1). So it can be assumed that lineage specific substitutions (associated with putative conformational remodeling and functional diversification) occurred at the expense of protein stability. These substitutions which were confined to the C-terminal have actually regulated the structural dynamics of the identified N-terminal region (32–58) which is termed as *“critical region”* of SNCA by the phenomena called as “*epistasis*”. The interaction study conducted between SNCA and coiled-coil domain of SNCAIP revealed that the critical region harbors crucial interaction sites which enlightened the vital role of this region in normal cellular as well as disease processes (Fig. 5a–d). The functional significance of this identified region was further advocated by various evidences reported previously. Rasia *et al*.^47^, stated an hypothesis that Cu(II) binding perturbs N-terminal long range interactions that are critical for stabilizing a soluble native like conformation of SNCA. Region 49–52 was reported as the strongest potential site for copper binding according to this study^47^. Recently, it was reported that interface for dopamine mediated SNCA dimerisation encompassed region 43–60 of SNCA^48^. These evidences strengthen our stated hypothesis that the critical region is not only performing its role in structural remodeling but also harbors crucial interaction sites for protein-protein or protein-metal interactions. It has been identified that SNCA mutants have a greater propensity to interact with and penetrate the lipid membrane through the domain from 36 to 45. This interaction causes an increase in SNCA oligomerization and toxicity which reinforces our assumption of the crucial role of this critical region in FPD pathogenesis^11^.

Although the normal cellular function and the metabolic pathways in which SNCA is involved are quite ambiguous, it has been reported that oxidative stress and proteasome inhibition are implicated in PD and other neurodegenerative disorders^49^,^50^. Apoptosis can be caused by proteasome inhibition and increased intracellular reactive oxygen species (ROS) levels can cause DNA and protein damage leading to cell death^51^. Various reports have shown that both proteasome inhibition and ROS can also trigger mitochondrial and endoplasmic reticulum (ER) stress cell death pathways^52^. PD patients have been observed to show increased levels of oxidative damage to DNA, lipids and proteins^53^. It has been accounted that oxidative stress increases SNCA aggregation. Oxidatively modified SNCA is more prone to aggregation than native protein. It has been identified that A53T mutant of SNCA causes increase in ER stress and elevate caspase-3, caspase-9 and caspase-12 activity^54^. A53T also causes mitochondrial depolarization resulting in accumulation of cytochrome c in cytosol leading to cell death^54^. Intriguingly, this human deleterious mutation (A53T) was discovered as a wild type in other sarcopterygian lineages analyzed i.e. non-hominoids, non-primate placental mammals, non-mammalian tetrapods (Table 1). These findings led us to hypothesize that the identified critical region plays a crucial mechanistic role in normal cellular functioning of SNCA. Furthermore, the reason behind observing “parkinsonism” only in humans can be attributed to human specific oxidative challenges and/or compensatory evolution in non-catarrhini sarcopterygians.

## Conclusion

Keeping in view the indispensable role of SNCA in the neurodegenerative processes, it is concluded that there are selective forces at work in sarcopterygians regulating the molecular and cellular mechanisms of SNCA. If this is the case, then fine tuning of these mechanisms through subtle changes in protein activity might be one of the contributing factors in bringing vital evolutionary changes to match the different environmental and ecological needs. The result of present study provides evidence that during the course of evolution, the region encompassed 32 to 58 of N-terminal lipid binding domain has acquired its critical significance in normal cellular function of SNCA and disease pathogenesis. The epistatic influence of the lineage specific substitutions might have led to the structural remodeling and functional innovation of SNCA. i.e., any mutation in this critical region is deleterious. These findings pave the way to investigate further the vital role of identified critical region of SNCA in different interaction studies and also with drug discovery perspective to target this region for the treatment of FPD.

## Additional Information

**How to cite this article**: Siddiqui, I. J. *et al*. The Parkinson Disease gene SNCA: Evolutionary and structural insights with pathological implication. *Sci. Rep*. **6**, 24475; doi: 10.1038/srep24475 (2016).

## Supplementary Material

Supplementary Information

Supplementary Data File

## Figures and Tables

**Table 1:** After the divergence from sarcopterygian ancestor, nine fixed amino-acid changes occurred at the root of mammals, whereas two occurred specifically in non-primate placental mammal’s lineage while one occurred specific in primate’s (catarrhini) lineage.

Position	Sarcopterygian Ancestral Residues	Replacement in Ancestor of Mammals	Non Primate Placental Mammals specific Replacements	Primates (catarrhini) specific Replacement	Neutral/ Radical	Impact on Protein Stability	Location
53	T			A^1^	Neutral (0)	Decreased (−0.71)	A_2_ Lipid binding alpha helix domain
64	S	T			Radical (+1)	Decreased (−0.26)	
68	G		E^1^		Radical (−2)	Decreased (−0.46)	
87	N	S			Radical (+1)	Decreased (−0.545)	NAC domain
94	L	F			Neutral (0)	Decreased (−0.508)	
95	V		G^1^		Radical (−3)	Decreased (−0.729)	
101	A	G			Neutral (0)	Decreased (−0.982)	
107	F	A			Radical (−2)	Decreased (−0.261)	C-Terminal acidic domain
112	M	I			Radical (+1)	Decreased (−0.81)	
113	M	L			Radical (+2)	Decreased (−0.01)	
129	P	S			Radical (−1)	Increased (0.487)	
132	E	G			Radical (−2)	Decreased (−0.514)	

This table represents the putative ancestral amino acid residues and the type of replacement occurred in each lineage since their divergence. The 6^th^ column depicts the putative physicochemical impact of each replacement on protein/structure function. The number within brackets are the log odds associated with changing the amino acids. Positive numbers imply a preferred change, zero implies a neutral change and negative numbers imply an un-preferred change. The 7^th^ column depicts the putative impact of each replacement on stability of protein. The number within brackets are the confidence scores (i.e. from -1 to 1) associated with the impact on stability of protein structure by changing the amino acids. Positive numbers imply an increase in stability of protein structure. The bigger the score, the more confident the prediction is. Conversely, negative numbers imply a decrease in the stability of protein structure.

^1^Represents the lineage specific substitutions in SNCA.

**Table 2:** Analysis of the lineage specific structural deviations in the backbone torsion angles of the ancestral SNCA proteins by incorporating the clade specific substitutions.

Comparison between lineages	Major change in backbone torsion angles (residue no)	Major shifts in region	Critical region
Primates ↔ Non Primate Placental Mammals	32–44,47–58	Lipid Binding Domain	(32–58) Lipid Binding Domain
	64–74	NAC domain	
	92–113	C-terminal domain	
Primates ↔ Mammalian Ancestor	37–58	Lipid Binding Domain	
	70	NAC domain	
Primates ↔ Sarcopterygian Ancestor	8–12,14,15,18,24,25, 29–47, 50–55, 58	Lipid Binding Domain	
	65–77	NAC domain	
	93–107, 109–140	C-terminal domain	

This table shows the effect of lineage specific substitutions on the backbone torsion angles of ancestral SNCA proteins by comparing with NMR structure of SNCA (1XQ8) (primates specific).
