# Supplementary material for: The Parkinson Disease gene SNCA: Evolutionary and structural insights with pathological implication
Source: Sci Rep. 2016 Apr 15;6:24475. doi: 10.1038/srep24475 (PMC4832246; doi:10.1038/srep24475)
Supplement: Supplementary Information [file srep24475-s1.pdf]

The Parkinson Disease gene SNCA: Evolutionary and structural insights with pathological implication

Irum Javaid Siddiqui, NashaimanPervaiz and Amir Ali Abbasi*

National Center for Bioinformatics, Program of Comparative and Evolutionary Genomics, Faculty of Biological Sciences, Quaid-i-Azam University, Islamabad 45320, Pakistan.

*Corresponding author

E-mail: abbasiam@qau.edu.pk

Tel Office:+92-51-90644109

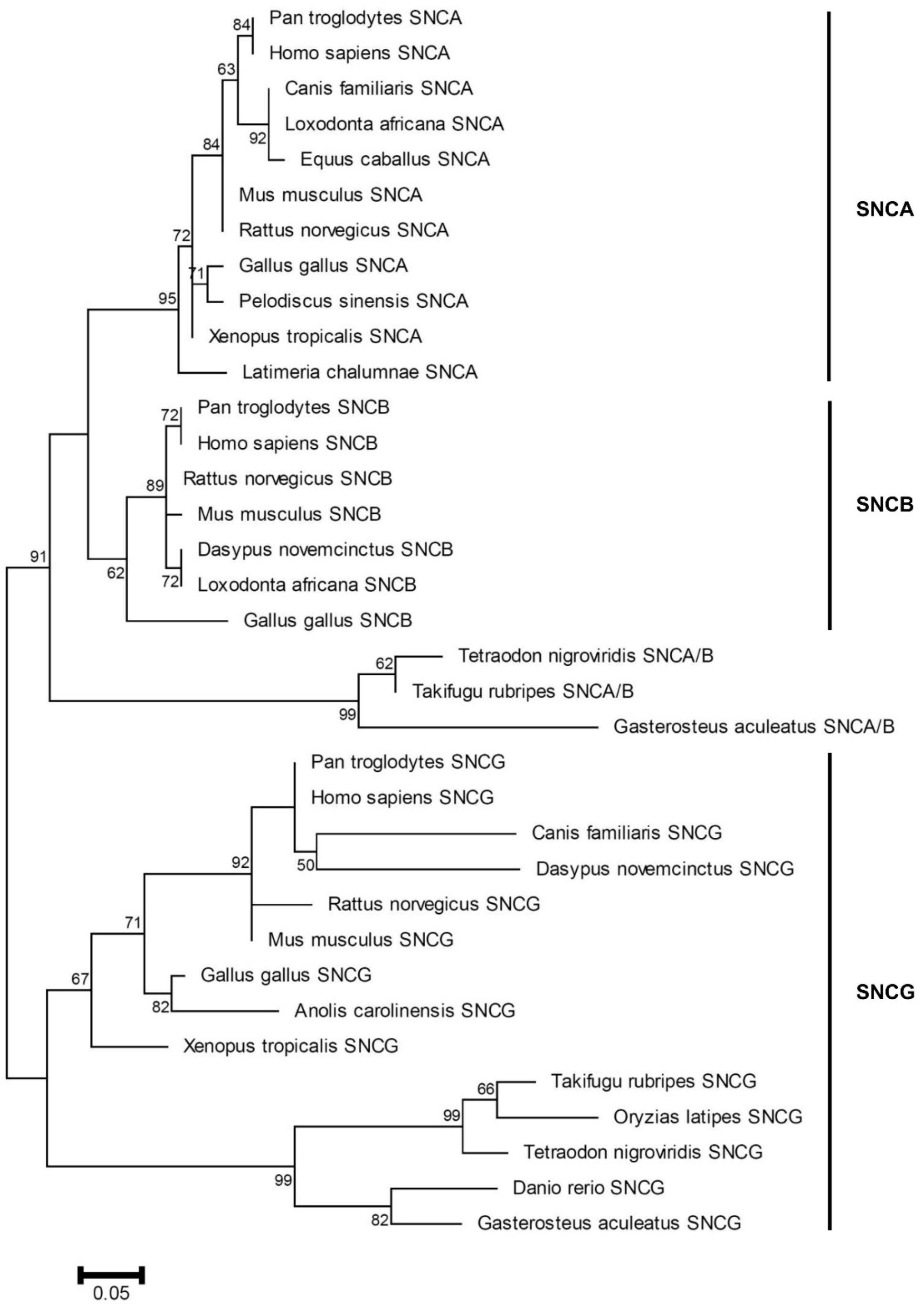

Figure S1. The evolutionary history was inferred by using the ML method based on the WAG model. The tree with the highest log likelihood (-1290.8345) is shown. The percentage of trees in which the associated taxa clustered together is shown next to the branches; only the values ≥ 50% are shown here. A discrete Gamma distribution was used to model evolutionary rate differences among sites (+G, parameter = 2.1877). The tree is drawn to scale, with branch lengths measured in the number of substitutions per site. The analysis involved 35 amino acid sequences. All positions containing gaps and missing data were eliminated. There were a total of 79 positions in the final dataset.

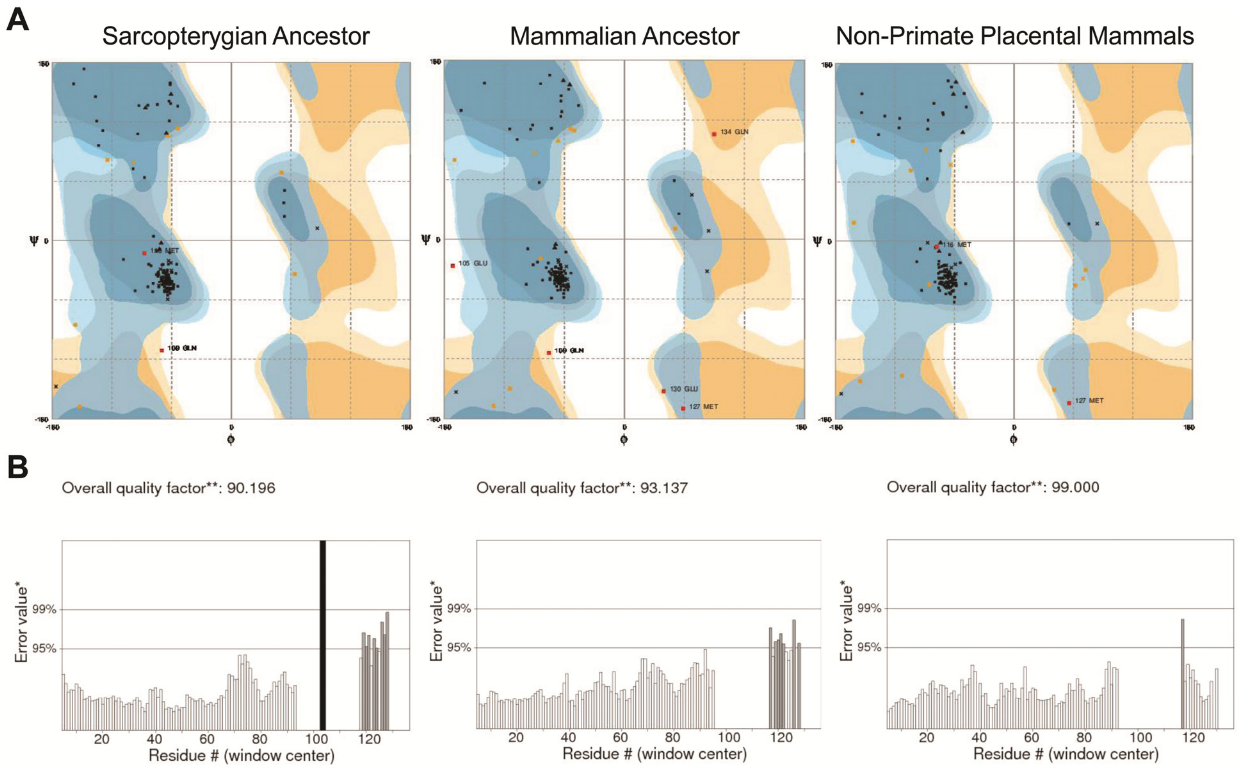

Figure S2. Evaluation of 3D models of SNCA proteins. (A) Ramachandran plots of sarcopterygian ancestral, mammalian ancestral and non-primate placental mammals specific SNCA proteins. **(B)** Quality of models have been examined with the help of Errat. Overall quality factor is expressed as percentage of the protein for which the calculated error value falls below the 95% rejection limit, calculated by Errat.

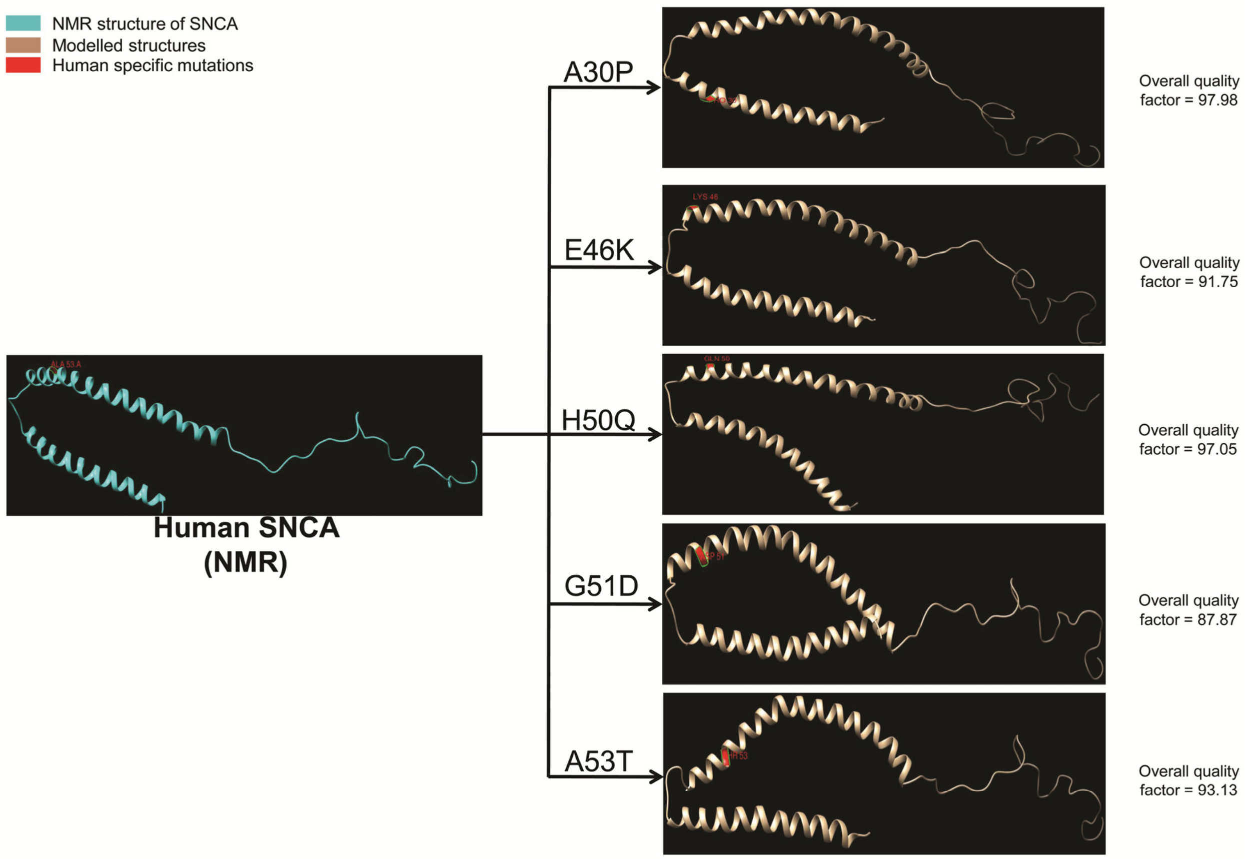

Figure S3. Structural analysis of mutant models of human SNCA generated from Modeller. Human specific mutations involved in FPD are red color coded. NMR structure of SNCA was obtained from PDB (1XQ8). Overall quality factor is expressed as percentage of the protein for which the calculated error value falls below the 95% rejection limit, calculated by Errat.

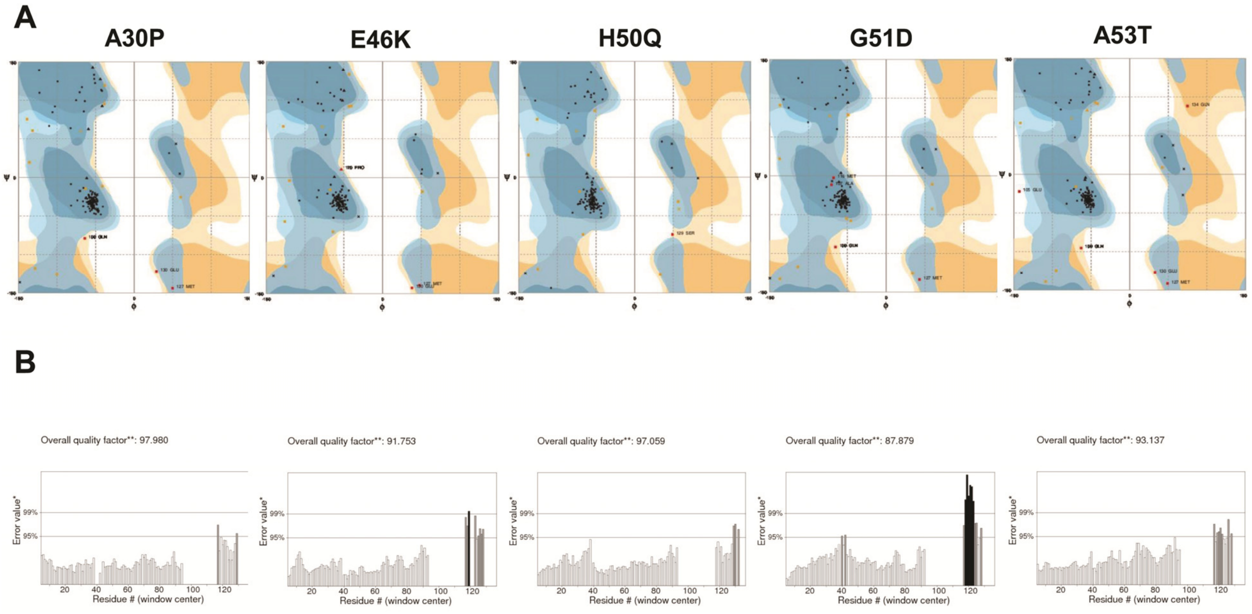

Figure S4. Evaluation of 3D models of mutant SNCA proteins. (A) Ramachandran plots of human specific SNCA mutations involved in FPD. **(B)** Quality of models have been examined with the help of Errat. Overall quality factor is expressed as percentage of the protein for which the calculated error value falls below the 95% rejection limit, calculated by Errat.

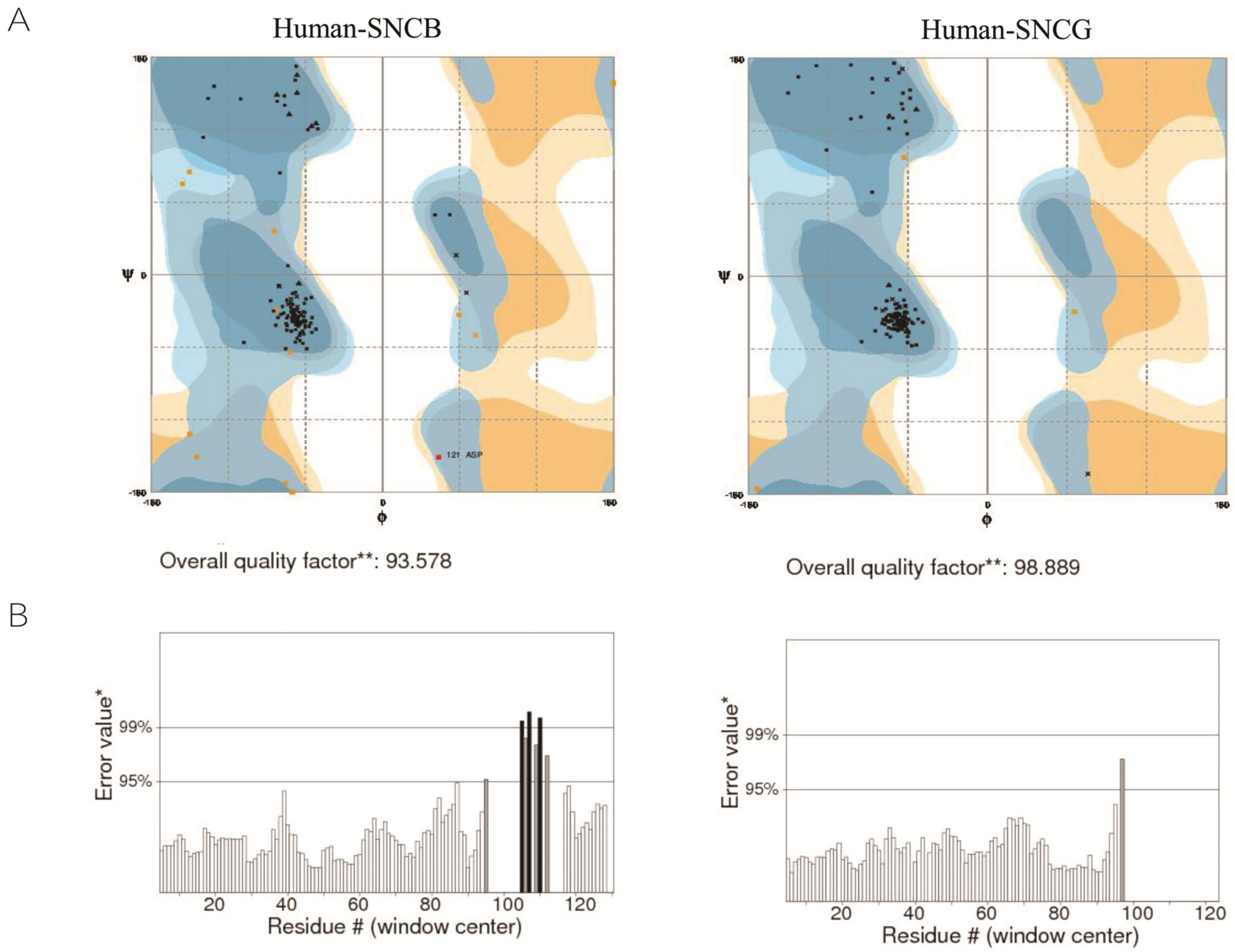

Figure S5. Evaluation of 3D models of human SNCB and human SNCG. (A) Ramachandran plots of human paralogous sequences, SNCB and SNCG. **(B)** Quality of models have been examined with the help of Errat. Overall quality factor is expressed as percentage of the protein for which the calculated error value falls below the 95% rejection limit, calculated by Errat.

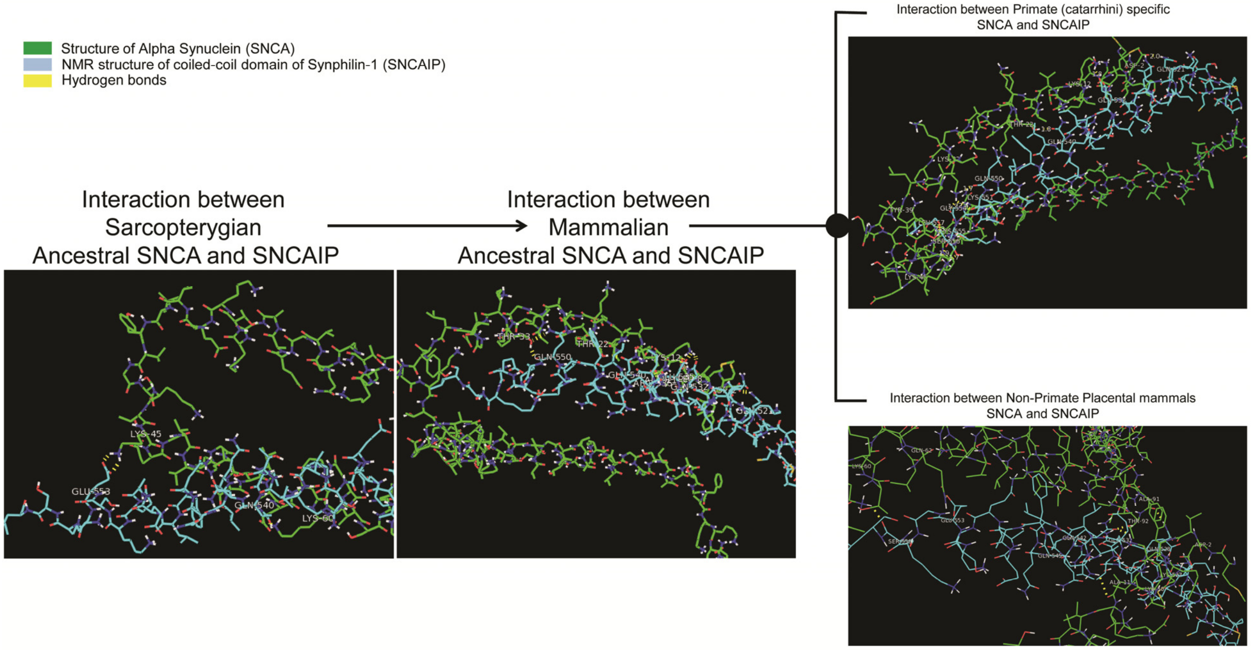

Figure S6. Analysis of the evolution of interactions between the ancestral SNCA proteins and coiled-coil domain of SNCAIP. Diagram displaying interactions between the sarcopterygian ancestral, mammalian ancestral, non-primate placental mammals specific and catarrhini's specific SNCA proteins and coiled-coil domain (510-557) of SNCAIP (2KES). SNCA proteins and SNCAIP interacting partner are color coded. Interacting residues are highlighted in white color. Hydrogen bonding is represented as dotted line in yellow.

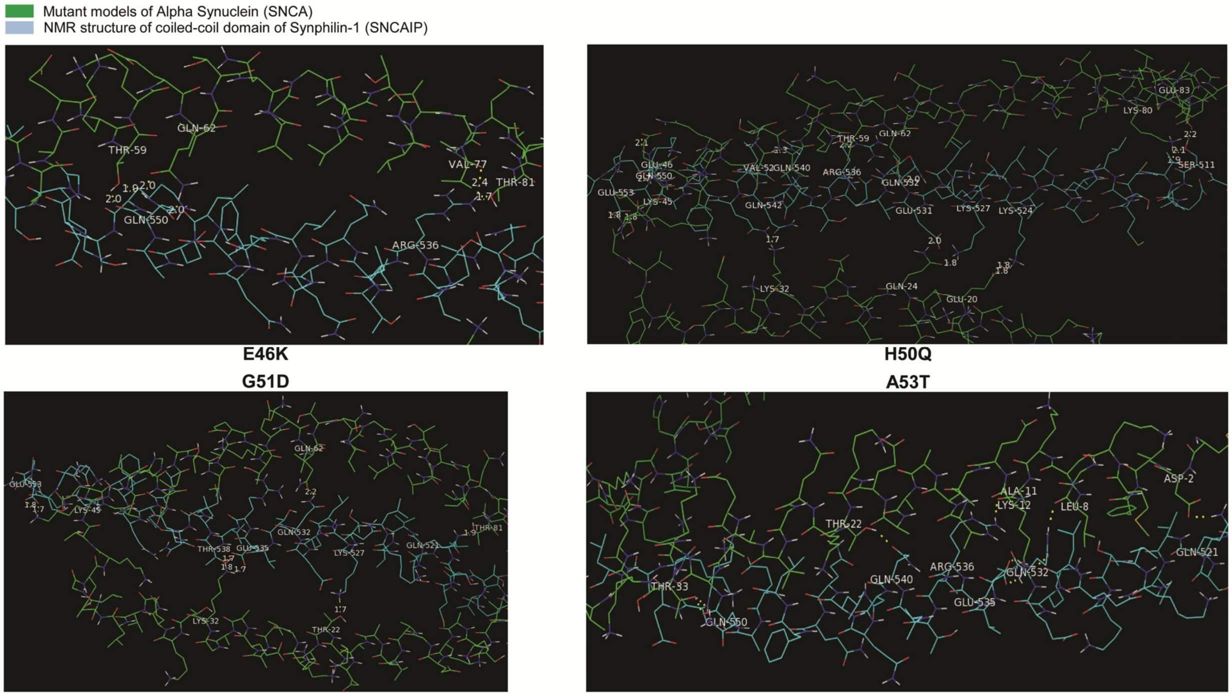

Figure S7. Analysis of the interacting residues involved in hydrogen bonding between the docked complexes of mutant models of SNCA and coiled-coil domain of SNCAIP. Diagrammatic view of the interactions between the mutant models of SNCA and coiled-coil domain (510-557) of SNCAIP (2KES). Mutant SNCA proteins and SNCAIP interacting partner are color coded. Interacting residues are highlighted in white color. Hydrogen bonding is represented as dotted line in yellow.

Table S1. Estimation of number of synonymous substitutions per synonymous site (dS), number of non-synonymous substitutions per non-synonymous site (dN) with the respective strength of the sequence (dN/dS) with Hyphy.

	dN/dS(dN,dS)	dN-dS[1]
**Substitution Model/Method →<br>Species-SNCA ↓**	**Codon Based (GY-94)**	
**Hominoids**		
Human	1 (1e-10,1e-10)	-0.05
Chimpanzee	1 (1e-10,1e-10)	
Gorilla	4 e-09 (1e-10,0.020)	
Orangutan	3 e-09 (1 e-10,0.031)	
**Non-Hominoids**		
Macaque	0.2 (0.02,0.097)	-1.969
Marmoset	0.08 (0.008,0.095)	
Squirrel Monkey	0.02 (0.017,0.842)	
Bushbaby	0.05 (0.057,1.037)	
**Non-Primate Placental Mammals**		
Mouse	0.02 (0.07,3.3)	-5.01
Dog	0.05 (0.02,0.36)	
Cow	0.04 (0.03,0.68)	
Elephant	0.01 (0.01,0.8)	
**Non-Mammalian Tetrapods**		
Chicken	0.03 (0.06,1.62)	-24.55
Turtle	0.4 (0.12,0.28)	
Frog	0.01 (0.13,8.91)	
Coelacanth	0.01 (0.26,14.31)	

[1]dN-dS<0 implies negative selection constraint on SNCA within sarcopterygian lineage.

Table S2. Estimation of number of synonymous substitutions per synonymous site (dS), number of non-synonymous substitutions per non-synonymous site (dN) with the respective strength of the sequence (dN/dS) with Hyphy.

	**dN/dS(dN,dS)**	**dN-dS[1]**
**Substitution Model/Method →<br>Species-SNCB ↓**	**Codon Based (GY-94)**	
**Hominoids**		
Human	0.07 (0.0014,0.018)	-0.04
Chimpanzee	1 (1e-010,1e-010)	
Gorilla	0.05 (0.001,0.018)	
Orangutan	0.07 (0.0007,0.009)	
**Non-Hominoids**		
Macaque	0.04 (0.006,0.133)	-1.40
Marmoset	0.35 (0.007,0.141)	
Squirrel Monkey	0.05 (0.002,0.04)	
Bushbaby	0.04 (0.05,1.47)	
**Non-Primate Placental Mammals**		
Mouse	0.13 (0.148,1.07)	-5.66
Dog	0.61 (0.02,0.18)	
Cow	0.12 (0.06,0.47)	
Elephant	0.13 (0.66,4.83)	
**Non-Mammalian Tetrapods**		
Chicken	0.05 (0.13,2.30)	-9.60
Turtle	0.05 (0.13,2.44)	
Frog	0.05 (0.10,1.82)	
Coelacanth	0.05 (0.20,3.61)	

[1]dN-dS<0 implies negative selection constraint on SNCB within sarcopterygian lineage.

Table S3. Estimation of number of synonymous substitutions per synonymous site (dS), number of non-synonymous substitutions per non-synonymous site (dN) with the respective strength of the sequence (dN/dS) with Hyphy.

	**dN/dS(dN,dS)**	**dN-dS[1]**
**Substitution Model/Method →<br>Species-SNCG ↓**	**Codon Based (GY-94)**	
**Hominoids**		
Human	0.83 (0.005,0.006)	-0.01
Chimpanzee	1 (1e-010,1e-010)	
Gorilla	0.8 (0.01,0.0125)	
Orangutan	0.86 (0.038,0.044)	
**Non-Hominoids**		
Macaque	0.2 (0.033,0.159)	-1.40
Marmoset	0.19 (0.015,0.076)	
Squirrel Monkey	0.2 (0.05,0.24)	
Bushbaby	0.2 (0.27,1.29)	
**Non-Primate Placental Mammals**		
Mouse	0.15 (0.223,1.478)	-4.82
Dog	0.15 (0.254,1.687)	
Cow	0.15 (0.131,0.869)	
Elephant	0.15 (0.248,1.647)	
**Non-Mammalian Tetrapods**		
Chicken	0.11 (0.378,3.425)	-22.21
Turtle	0.11 (0.085,0.77)	
Frog	0.11 (0.428,3.88)	
Coelacanth	0.11 (1.866,16.9)	
**Fishes**		
Fugu	0.18 (0.164,0.87)	-1.92
Tetraodon	0.18 (0.03,0.162)	
Stickleback	0.18 (0.143,0.76)	
Medaka	0.18 (0.11,0.58)	

[1]dN-dS<0 implies negative selection constraint on SNCG within sarcopterygian lineage.

Table S4. Identification of negatively constrained sites in SNCA among sarcopterygians at 0.1 significance level with Hyphy.

Index	Residue no	dN-dS	P-value
1	12	-2.860705	0.06
2	13	-2.860572	0.06
3	15	-3	0.03
4	18	-4	0.01
5	20	-4.272426	0.01
6	23	-7.122523	0.0009
7	30	-3	0.03
8	37	-5	0.004
9	39	-4.83201	0.02
10	41	-3	0.03
11	46	-2.848435	0.06
12	47	-4	0.01
13	49	-4	0.01
14	50	-4.177328	0.05
15	52	-3	0.03
16	62	-4.282012	0.01
17	65	-4.83201	0.01
18	67	-5	0.004
19	69	-6	0.001
20	72	-4	0.01
21	73	-5	0.004
22	75	-3	0.03
23	77	-4	0.01
24	78	-4	0.01
25	83	-2.854275	0.06
26	85	-3	0.03
27	86	-3	0.03
28	88	-2.894755	0.05
29	94	-3.150189	0.07
30	98	-7.248015	0.002

*Abbreviations:*dS, synonymous substitutions per synonymous site; dN, non-synonymous substitutions per non-synonymous site.4th column depicts p-value ($p<0.1$) suggesting putative negatively constrained sites.

Table S5: Analysis of the interacting residues involved in hydrogen bonding between the clades specific docked complexes of SNCA and coiled-coil domain of SNCAIP.

Docked complex	Binding energy (kcal/mol)	Interacting residues of SNCA	Interacting residues of SNCAIP	Hydrogen Bonding (Å)
**Primates (Catarrhini) SNCA-SNCAIP complex**	-805.8	Asp2[a]	Gln521	2
		Lys12[a]	Gln532	1.8
		Thr22[a]	Gln540	1.8
		Lys32[b]	Gln550	1.7
		Lys32[b]	Lys551	2.4
		Lys32[b]	Gly554	1.7
		Tyr39[b]	Leu557	2.3
		Lys45[b]	Lys555	1.9
		Lys45[b]	Ser556	1.7
**Non-primate placental mammals SNCA-SNCAIP complex**	-779.4	Asp2	Lys527	1.7
		Lys10	Gln542	1.7
		Ala11	Gln545	2.6
		Lys60	Ser556	1.7
		Gly62	Glu553	2
		Ala91	Gln528	2
		Thr92	Gln532	2
**Mammalian Ancestral SNCA-SNCAIP complex**	-767.9	Asp2[a]	Gln521	2.1
		Leu8	Gln532	2
		Ala11	Arg536	1.8
		Lys12	Glu535	1.7
		Lys12	Glu535	1.8
		Lys12[a]	Gln532	1.7
		Thr22[a]	Gln540	1.9
		Thr33	Gln550	2.2
		Lys45	Glu553	1.8
**Sarcopterygian Ancestral SNCA-SNCAIP complex**	-722.5	Lys45	Glu553	1.8
		Lys60	Gln540	1.9
		Lys60	Gln540	1.9

This table depicts interactions between the ancestral SNCA proteins and coiled-coil domain (510-557) of SNCAIP (2KES). 2nd column depicts the lowest binding energies of the docked complexes in kcal/mol. 4th column represents hydrogen bonding between the hydrogen bond donor and hydrogen bond acceptor (Å) in docked complexes.
[a]Interactions conserved between mammalian ancestor and catarrhines.
[b]Interactions that transpired specific to catarrhines.

Table S6: Analysis of the interacting residues involved in hydrogen bonding between the docked complexes of mutant models of SNCA and coiled-coil domain of SNCAIP.

Docked Complex	Binding energy (kcal/mol)	Interacting residues of SNCA	Interacting residues of SNCAIP	Hydrogen Bonding (Å)
**A30P-SNCAIP complex**	-744.8	Thr81	Gln532	2.5
		Thr81	Arg536	1.7
		Thr81	Arg536	2.1
		Gly84	Gln540	2.3
		Ser87	Gln540	2
		Thr92	Gln542	2.1
		Val95	Gln550	2.2
		Lys97	Glu553	1.6
		Lys97	Gln550	1.8
**E46K-SNCAIP complex**	-779.1	Thr59	Gln550	2
		Thr59	Gln550	1.9
		Gln62	Gln550	2
		Gln62	Gln550	2
		Val77	Arg536	2.4
		Thr81	Arg536	1.7
**H50Q-SNCAIP complex**	-703.2	Glu20	Lys524	1.8
		Glu20	Lys524	1.8
		Gln24	Lys527	1.8
		Gln24	Glu531	2
		Lys32	Gln542	1.7
		Lys45	Gln553	1.8
		Lys45	Glu553	1.8
		Glu46	Gln550	2.1
		Glu46	Gln550	2.7
		Val52	Gln540	2.3
		Thr59	Arg536	2.2
		Gln62	Gln532	2
		Lys80	Ser511	1.9
		Lys80	Ser511	1.9
		Glu83	Ser511	2.2
		Lys32	Glu535	1.7
		Lys32	Glu535	1.7
		Lys32	Thr538	1.8

		Lys45	Glu553	1.7
		Lys45	Glu553	1.8
		Gln62	Gln532	2.2
		Thr81	Gln521	1.9
**A53T-SNCAIP complex**	-767.9	Asp2	Gln521	2.1
		Leu8	Gln532	2
		Ala11	Arg536	1.8
		Lys12	Gln532	1.7
		Lys12	Glu535	1.7
		Lys12	Glu535	1.8
		Thr22	Gln540	1.9
		Thr33	Gln550	2.2
		Thr33	Gln550	1.9
		Thr33	Gln550	1.9

This table depicts interactions (in terms of hydrogen bonding) between mutant models of SNCA proteins and the coiled-coil domain of SNCAIP (510-557). 2nd column depicts the lowest binding energies of the docked complexes in kcal/mol. 4th column represents hydrogen bonding between the hydrogen bond donor and hydrogen bond acceptor (Å) in docked complexes.
