# Supplementary material for: The Parkinson Disease gene SNCA: Evolutionary and structural insights with pathological implication
Source: Sci Rep. 2016 Apr 15;6:24475. doi: 10.1038/srep24475 (PMC4832246; doi:10.1038/srep24475)
Supplement: Supplementary Data File [file srep24475-s2.doc]

**Complete list of protein sequences used in this study**

**SYNUCLEIN FAMILY-NJ**

>Homo sapiens_SNCA (ENSP_338345)

MDVFMKGLSKAKEGVVAAAEKTKQGVAEAAGKTKEGVLYVGSKTKEGVVHGVATVAEKTKEQVTNVGGAVVTGVTAVAQKTVEGAGSIAAATGFVKKDQLGKNEEGAPQEGILEDMPVDPDNEAYEMPSEEGYQDYEPEA

>Pan troglodytes_SNCA (ENSPTRP_54542)

MDVFMKGLSKAKEGVVAAAEKTKQGVAEAAGKTKEGVLYVGSKTKEGVVHGVATVAEKTKEQVTNVGGAVVTGVTAVAQKTVEGAGSIAAATGFVKKDQLGKNEEGAPQEGILEDMPVDPDNEAYEMPSEEGYQDYEPEA

>Mus musculus_SNCA (ENSMUSP_109907)

MDVFMKGLSKAKEGVVAAAEKTKQGVAEAAGKTKEGVLYVGSKTKEGVVHGVTTVAEKTKEQVTNVGGAVVTGVTAVAQKTVEGAGNIAAATGFVKKDQMGKGEEGYPQEGILEDMPVDPGSEAYEMPSEEGYQDYEPEA

>Rattus norvegicus_SNCA (ENSRNOP_30609)

MDVFMKGLSKAKEGVVAAAEKTKQGVAEAAGKTKEGVLYVGSKTKEGVVHGVTTVAEKTKEQVTNVGGAVVTGVTAVAQKTVEGAGNIAAATGFVKKDQMGKGEEGYPQEGILEDMPVDPSSEAYEMPSEEGYQDYEPEA

>Canis familiaris_SNCA (ENSCAFP_14518)

MDVFMKGLSKAKEGVVAAAEKTKQGVAEAAGKTKEGVLYVGSKTKEGVVHGVTTVAEKTKEQVTNVGEAVVTGVTAVAQKTVEGAGSIAAATGFGKKDQLGKSEEGGPQEGILEDMPVDPDNEAYEMPSEEGYQDYEPEA

>Equus caballus_SNCA (ENSECAT_16509)

MDVFMKGLSKAKEGVVAAAEKTKQGVAEAAGKTKEGVLYVGSKTKEGVVHGVTTVAEKTKEQVTNVGEAVVTGVTAVAQKTVEGAESIAAATGFGKKDHLGKSEEGAAQEGILEDMPVDPDNEAYEMPSEEGYQDYEPEA

>Loxodonta africana_SNCA (ENSLAFP_9764)

MDVFMKGLSKAKEGVVAAAEKTKQGVAEAAGKTKEGVLYVGSKTKEGVVHGVTTVAEKTKEQVTNVGEAVVTGVTAVAQKTVEGAGSIAAATGFGKKDQMGKGEEGAPQEGILENVPVDPDNEAYEMPSEEGYQDYEPEA

>Gallus gallus_SNCA (ENSGALP_16889)

MDVFMKGLNKAKEGVVAAAEKTKQGVAEAAGKTKEGVLYVGSRTKEGVVHGVTTVAEKTKEQVSNVGGAVVTGVTAVAQKTVEGAGNIAAATGLVKKDQLAKQNEEGFLQEGMVNNTDIPVDPENEAYEMPPEEEYQDYEPEA

>Pelodiscus sinensis_SNCA (ENSPSIP_12308)

MDVFMKGLSKAKEGVVAAAEKTKQGMAEAAGKTKEGVLYVGSRTREGVVHGVTTMAEKTKEQVSNVGGAVVTGVTAVAHKTVEGAGNIAAATGLVKKDQMAKQNEEGLSQEGMMDSTDMPMDPDNEAYEMPPEEEYQDYEPEA

>Xenopus tropicalis_SNCA (ENSXETP_51005)

MDVFMKGLSKAKEGVVAAAEKTKQGVAEAAGKTKEGVLYVGSKTKEGVVHGVTTVAEKTKEQVSNVGGAVVTGVTAVAQKTVEGAGNIAAATGLVKKDQK

>Latimeria chalumnae_SNCA (ENSLACP_13985)

MDMLMKGLSKAKEGVVAAAEKTKQGVAEAAGKTKEGVLYMGSKTKEGVVQGVTTVAEKTKEQVSNVGGAVVTGVTAVAQKTVEGAGNIAAATGLVKKDQLNKE

>Takifugu rubripes_SNCA (ENSTRUP_24283)

MDAFMKGFSKAKDGVVAAAEKTKQGVTGAAEMTKDGVMFVGTKTKDGVTVVAGKTVSGVSQVGGAMVTGVTAVAQKTVESAGSIAAATGLVKKEPGKQGDDAATAENMAESPDVTDPAEATEEDADD

>Tetraodon nigroviridis_SNCA (ENSTNIP_3705)

MDALMKGFSKAKDGVVAAAEKTKQGVTGAAEMTKDGVMFVGTKTKDGVTVAVAGKTVSGMSQVGGAVVTGVTTVAQKTVESAGSIAAATGLVKKEPGKQSDGTDGPAPEDPDGSPDVTDPGQATEEDSDD

>Homo sapiens_SNCB (ENSP_308057)

MDVFMKGLSMAKEGVVAAAEKTKQGVTEAAEKTKEGVLYVGSKTREGVVQGVASVAEKTKEQASHLGGAVFSGAGNIAAATGLVKREEFPTDLKPEEVAQEAAEEPLIEPLMEPEGESYEDPPQEEYQEYEPEA

>Pan troglodytes__SNCB ([ENSPTRP_30003](http://asia.ensembl.org/Pan_troglodytes/Transcript/ProteinSummary?db=core;g=ENSPTRG00000002708;r=10:86958618-86963260;t=ENSPTRP00000030003;tl=J5XZvmmjrkThIOhZ-639172-114613573))

MDVFMKGLSMAKEGVVAAAEKTKQGVTEAAEKTKEGVLYVGSKTREGVVQGVASVAEKTKEQASHLGGAVFSGAGNIAAATGLVKREEFPTDLKPEEVAQEAAEEPLIEPLMEPEGESYEDPPQEEYQEYEPEA

>Rattus norvegicus_SNCB (ENSRNOP_24357)

MDVFMKGLSMAKEGVVAAAEKTKQGVTEAAEKTKEGVLYVGSKTKEGVVQGVASVAEKTKEQASHLGGAVFSGAGNIAAATGLVKKEEFPTDLKPEEVAQEAAEEPLIEPLMEPEGESYEDSPQEEYQEYEPEA

>Loxodonta africana_SNCB (ENSLAFP_16651)

MDMFMKGLSMAKEGVVAAAEKTKQGVTEAAEKTKEGVLYVGSKTREGVVQGVASAEKTKEQASHLGGAVFSGAGNIAAATGLVKKEEFPTDLKDMFPQTMATQSCEQPISVPRRSCDPRCVGEVYHDNRRQRYQEYEPERKGP

>Dasypus novemcinctus_SNCB (ENSDNOP_30923)

MDMFMKGLSMAKEGVVAAAEKTKQGVTEAAEKTKEGVLYVGSKTREGVVQGVASVAEKTKEQASHLGGAVFSGAGNIAAATGLVKKEEFPTDLKPEEVAQEAAEEPLIEPLMEPEGESYEEPLQEEYQEYEPEA

>Gallus gallus_SNCB (ENSGALP_40085)

MEVFMKGLSKAKEGVVAAAEKTKQGVAEAAEKTKEGVLYVGSKTQGVVQGVTSVAEKAKEQASQLGEAAFSGAGNIAAATGLVKKEEFPADLKAEEVAQEAVEEPLVEPLLEPEGESYEESPQEEYQEYEPEA

>Homo sapiens_SNCG (ENSP_361087)

MDVFKKGFSIAKEGVVGAVEKTKQGVTEAAEKTKEGVMYVGAKTKENVVQSVTSVAEKTKEQANAVSEAVVSSVNTVATKTVEEAENIAVTSGVVRKEDLRPSAPQQEGEASKEKEEVAEEAQSGGD

>Pan troglodytes_SNCG (ENSPTRP_4729)

MDVFKKGFSIAKEGVVGAVEKTKQGVTEAAEKTKEGVMYVGAKTKENVVQSVTSVAEKTKEQANAVSEAVVSSVNTVATKTVEEAENIAVTSGVVRKEDLKPSAPQQEGEASKEKEEVAEEAQSGGD

>Mus musculus_SNCG (ENSMUSP_23826)

MDVFKKGFSIAKEGVVGAVEKTKQGVTEAAEKTKEGVMYVGTKTKENVVQSVTSVAEKTKEQANAVSEAVVSSVNTVANKTVEEAENIVVTTGVVRKEDLEPPAQDQEAKEQEENEEAKSGED

>Rattus norvegicus_SNCG (ENSRNOP_63571)

MDVFKKGFSIAREGVVGAVEKTKQGVTEAAEKTKEGVMYVGTKTKERGTSVTSVAEKTKEQANAVSEAVVSSVNTVATKTVEEAENIVVTTGVVRKEDLEPPAQDQEAKEQEEGEEAKSGGD

>Canis familiaris_SNCG (XP_005619498)

MGCLGWASEVALGAGKASPSQEGVVGAVEKTKQGVTEAAEKTKEGVLYVGAKTKENVVQSVTSVAEKTKEQANAVSEAVVTSINTVAVKTVEEAENIAITSGVVRKEDLEQPAAPQEDKAARVQEEVAEEAKSGGD

>Gallus gallus_SNCG (ENSGALP_3131)

MDVFKKGFSIAKEGVVAAAEKTKQGVTEAAEKTKEGVMYVGTKTKEGVVQSVTSVAEKTKEQANVVGEAVVASVNTVANKTVEGAETIVATTGVVKKEDLAPQQPAAEGEAAIPGSTEGGGEGENEGN

>Anolis carolinensis_SNCG (ENSACAP_14476)

MDVFKKGFSIAKEGVVAAAEKTKQGVTEAAEKTKEGVMYVGAKTKEGVVQSVTSVAEKTKEQANLVGESVVASVNTVAKQTVEGAETVVSSTGVVKMEDLHPEQPEEPLADAEEDAPVEATETSPEGENEGY

>Xenopus tropicalis_SNCG (ENSXETP_7895)

MDVFKKGFSMAKEGVVAAAEKTKQGVTEAAEKTKEGVMYVGAKTKEGVVHSVSTVAEKTKEQANVVGGAVVSGVNQVASKTVEGTENIVGTTGLVKKEDLHPGQPEEPAAEEEPAVEATESTEQVGDGEN

>Danio rerio_SNCG (ENSDARP_123611)

MDVLMKGFSMAKEGVVAAAEKTKAGVEEAALKTKEGVMYVGNKTKEGMVSGVNTVVQKTTDQANIVGETAVGGANVAGQNTVEGLENVAASTGMVNPGDFSHGGMEGGEGGEGY

>Takifugu rubripes_SNCG (ENSORLP_13076)

MDVLKKGFSMAKDGVVAAAEKTKAGVEGAATKTKEGVIYVGNKTMEGVVTSVNTVAHKTTEQANIIADTAVSGANEVAQSAVEGVENAAVASGLVSLEEAGPVSEKAGVPNTEAEAEESEQVVQ

>Tetraodon nigroviridis_SNCG (ENSTNIP_4769)

MDALKKGFSMAKGGVVAAAEKTKAGVEEAATKTKEGVIYVGNKTMEGVVTGVNTVAHKTTEQANIIADTAVAGANEVAQSAVEGVENAAMASGLVKKEEAGPESEKAEAPGPEAEEEQSKQAAQ

>Gasterosteus aculeatus_SNCG (ENSGACP_3738)

MDVFMKGFSMAKEGVVAAAEKTKAGMEEAAAKTKEGVMYVGSKTKEGVVSSVNTVANRTVDQANIVGDTAVAGANEVSQAAVEGVENVAASTGMVNQGEYGGMEQGGEGGEGY

>Oryzias latipes_SNCG (ENSORLP_3349)

MDVLKKGFSMAKDGVVAAAEKTKAGVEEAATKTKEGVIYVGNKTMEGVVTGVNTVAQKSTEQVNVAAETAVAGANEVAEATVEGVENAAVASGFVSMEEAGPVAAEADLPKTAAGGEQNQPAAE

**Complete list of protein sequences used in this study**

**SYNUCLEIN FAMILY-ML**

>Homo sapiens_SNCA (ENSP_338345)

MDVFMKGLSKAKEGVVAAAEKTKQGVAEAAGKTKEGVLYVGSKTKEGVVHGVATVAEKTKEQVTNVGGAVVTGVTAVAQKTVEGAGSIAAATGFVKKDQLGKNEEGAPQEGILEDMPVDPDNEAYEMPSEEGYQDYEPEA

>Pan troglodytes_SNCA (ENSPTRP_54542)

MDVFMKGLSKAKEGVVAAAEKTKQGVAEAAGKTKEGVLYVGSKTKEGVVHGVATVAEKTKEQVTNVGGAVVTGVTAVAQKTVEGAGSIAAATGFVKKDQLGKNEEGAPQEGILEDMPVDPDNEAYEMPSEEGYQDYEPEA

>Mus musculus_SNCA (ENSMUSP_109907)

MDVFMKGLSKAKEGVVAAAEKTKQGVAEAAGKTKEGVLYVGSKTKEGVVHGVTTVAEKTKEQVTNVGGAVVTGVTAVAQKTVEGAGNIAAATGFVKKDQMGKGEEGYPQEGILEDMPVDPGSEAYEMPSEEGYQDYEPEA

>Rattus norvegicus_SNCA (ENSRNOP_30609)

MDVFMKGLSKAKEGVVAAAEKTKQGVAEAAGKTKEGVLYVGSKTKEGVVHGVTTVAEKTKEQVTNVGGAVVTGVTAVAQKTVEGAGNIAAATGFVKKDQMGKGEEGYPQEGILEDMPVDPSSEAYEMPSEEGYQDYEPEA

>Canis familiaris_SNCA (ENSCAFP_14518)

MDVFMKGLSKAKEGVVAAAEKTKQGVAEAAGKTKEGVLYVGSKTKEGVVHGVTTVAEKTKEQVTNVGEAVVTGVTAVAQKTVEGAGSIAAATGFGKKDQLGKSEEGGPQEGILEDMPVDPDNEAYEMPSEEGYQDYEPEA

>Equus caballus_SNCA (ENSECAT_16509)

MDVFMKGLSKAKEGVVAAAEKTKQGVAEAAGKTKEGVLYVGSKTKEGVVHGVTTVAEKTKEQVTNVGEAVVTGVTAVAQKTVEGAESIAAATGFGKKDHLGKSEEGAAQEGILEDMPVDPDNEAYEMPSEEGYQDYEPEA

>Loxodonta africana_SNCA (ENSLAFP_9764)

MDVFMKGLSKAKEGVVAAAEKTKQGVAEAAGKTKEGVLYVGSKTKEGVVHGVTTVAEKTKEQVTNVGEAVVTGVTAVAQKTVEGAGSIAAATGFGKKDQMGKGEEGAPQEGILENVPVDPDNEAYEMPSEEGYQDYEPEA

>Gallus gallus_SNCA (ENSGALP_16889)

MDVFMKGLNKAKEGVVAAAEKTKQGVAEAAGKTKEGVLYVGSRTKEGVVHGVTTVAEKTKEQVSNVGGAVVTGVTAVAQKTVEGAGNIAAATGLVKKDQLAKQNEEGFLQEGMVNNTDIPVDPENEAYEMPPEEEYQDYEPEA

>Pelodiscus sinensis_SNCA (ENSPSIP_12308)

MDVFMKGLSKAKEGVVAAAEKTKQGMAEAAGKTKEGVLYVGSRTREGVVHGVTTMAEKTKEQVSNVGGAVVTGVTAVAHKTVEGAGNIAAATGLVKKDQMAKQNEEGLSQEGMMDSTDMPMDPDNEAYEMPPEEEYQDYEPEA

>Xenopus tropicalis_SNCA (ENSXETP_51005)

MDVFMKGLSKAKEGVVAAAEKTKQGVAEAAGKTKEGVLYVGSKTKEGVVHGVTTVAEKTKEQVSNVGGAVVTGVTAVAQKTVEGAGNIAAATGLVKKDQK

>Latimeria chalumnae_SNCA (ENSLACP_13985)

MDMLMKGLSKAKEGVVAAAEKTKQGVAEAAGKTKEGVLYMGSKTKEGVVQGVTTVAEKTKEQVSNVGGAVVTGVTAVAQKTVEGAGNIAAATGLVKKDQLNKE

>Takifugu rubripes_SNCA (ENSTRUP_24283)

MDAFMKGFSKAKDGVVAAAEKTKQGVTGAAEMTKDGVMFVGTKTKDGVTVVAGKTVSGVSQVGGAMVTGVTAVAQKTVESAGSIAAATGLVKKEPGKQGDDAATAENMAESPDVTDPAEATEEDADD

>Tetraodon nigroviridis_SNCA (ENSTNIP_3705)

MDALMKGFSKAKDGVVAAAEKTKQGVTGAAEMTKDGVMFVGTKTKDGVTVAVAGKTVSGMSQVGGAVVTGVTTVAQKTVESAGSIAAATGLVKKEPGKQSDGTDGPAPEDPDGSPDVTDPGQATEEDSDD

>Gasterosteus aculeatus_SNCA (ENSGACP_21837)

MDAFKKGFSKARDGVAAVAEKTKQGVTGAAEMTKDGVMFVGNKTKDGVTTDFPTAVSGVSQVGGAMVTGVTAVAHKTVEGAGNMVVATGLVKKDPAKQSDEASAVQDMAESPVDTDPADAMEDDADDN

>Homo sapiens__SNCB (ENSP_308057)

MDVFMKGLSMAKEGVVAAAEKTKQGVTEAAEKTKEGVLYVGSKTREGVVQGVASVAEKTKEQASHLGGAVFSGAGNIAAATGLVKREEFPTDLKPEEVAQEAAEEPLIEPLMEPEGESYEDPPQEEYQEYEPEA

>Pan troglodytes__SNCB ([ENSPTRP_30003](http://asia.ensembl.org/Pan_troglodytes/Transcript/ProteinSummary?db=core;g=ENSPTRG00000002708;r=10:86958618-86963260;t=ENSPTRP00000030003;tl=J5XZvmmjrkThIOhZ-639172-114613573))

MDVFMKGLSMAKEGVVAAAEKTKQGVTEAAEKTKEGVLYVGSKTREGVVQGVASVAEKTKEQASHLGGAVFSGAGNIAAATGLVKREEFPTDLKPEEVAQEAAEEPLIEPLMEPEGESYEDPPQEEYQEYEPEA

>Mus musculus_SNCB (ENSMUSP_116296)

MDVFMKGLSMAKEGVVAAAEKTKQGVTEAAEKTKEGVLYVGSKTSGVVQGVASVAEKTKEQASHLGGAVFSGAGNIAAATGLVKKEEFPTDLKPEEVAQEAAEEPLIEPLMEPEGESYEDSPQEEYQEYEPEA

>Rattus norvegicus_SNCB (ENSRNOP_24357)

MDVFMKGLSMAKEGVVAAAEKTKQGVTEAAEKTKEGVLYVGSKTKEGVVQGVASVAEKTKEQASHLGGAVFSGAGNIAAATGLVKKEEFPTDLKPEEVAQEAAEEPLIEPLMEPEGESYEDSPQEEYQEYEPEA

>Loxodonta africana_SNCB (ENSLAFP_16651)

MDMFMKGLSMAKEGVVAAAEKTKQGVTEAAEKTKEGVLYVGSKTREGVVQGVASAEKTKEQASHLGGAVFSGAGNIAAATGLVKKEEFPTDLKDMFPQTMATQSCEQPISVPRRSCDPRCVGEVYHDNRRQRYQEYEPERKGP

>Dasypus novemcinctus_SNCB (ENSDNOP_30923)

MDMFMKGLSMAKEGVVAAAEKTKQGVTEAAEKTKEGVLYVGSKTREGVVQGVASVAEKTKEQASHLGGAVFSGAGNIAAATGLVKKEEFPTDLKPEEVAQEAAEEPLIEPLMEPEGESYEEPLQEEYQEYEPEA

>Gallus gallus_SNCB (ENSGALP_40085)

MEVFMKGLSKAKEGVVAAAEKTKQGVAEAAEKTKEGVLYVGSKTQGVVQGVTSVAEKAKEQASQLGEAAFSGAGNIAAATGLVKKEEFPADLKAEEVAQEAVEEPLVEPLLEPEGESYEESPQEEYQEYEPEA

>Homo sapiens_SNCG (ENSP_361087)

MDVFKKGFSIAKEGVVGAVEKTKQGVTEAAEKTKEGVMYVGAKTKENVVQSVTSVAEKTKEQANAVSEAVVSSVNTVATKTVEEAENIAVTSGVVRKEDLRPSAPQQEGEASKEKEEVAEEAQSGGD

>Pan troglodytes_SNCG (ENSPTRP_4729)

MDVFKKGFSIAKEGVVGAVEKTKQGVTEAAEKTKEGVMYVGAKTKENVVQSVTSVAEKTKEQANAVSEAVVSSVNTVATKTVEEAENIAVTSGVVRKEDLKPSAPQQEGEASKEKEEVAEEAQSGGD

>Mus musculus_SNCG (ENSMUSP_23826)

MDVFKKGFSIAKEGVVGAVEKTKQGVTEAAEKTKEGVMYVGTKTKENVVQSVTSVAEKTKEQANAVSEAVVSSVNTVANKTVEEAENIVVTTGVVRKEDLEPPAQDQEAKEQEENEEAKSGED

>Rattus norvegicus_SNCG (ENSRNOP_63571)

MDVFKKGFSIAREGVVGAVEKTKQGVTEAAEKTKEGVMYVGTKTKERGTSVTSVAEKTKEQANAVSEAVVSSVNTVATKTVEEAENIVVTTGVVRKEDLEPPAQDQEAKEQEEGEEAKSGGD

>Canis familiaris_SNCG (XP_005619498)

MGCLGWASEVALGAGKASPSQEGVVGAVEKTKQGVTEAAEKTKEGVLYVGAKTKENVVQSVTSVAEKTKEQANAVSEAVVTSINTVAVKTVEEAENIAITSGVVRKEDLEQPAAPQEDKAARVQEEVAEEAKSGGD

>Dasypus novemcinctus_SNCG (ENSDNOP_27110)

MDIVRKGLSIAREGVAGAAEKTKQGVTEAAEKTKQGVTEAAEKTKQGVMYVGAKTKEGVVQSVTSVAEKTREQAAAVSDAVVTSVNSVATKTVEEAENIALTAGVVRKESLVPPAPPQEDEAATEEEAGAEEAKTEGD

>Gallus gallus_SNCG (ENSGALP_3131)

MDVFKKGFSIAKEGVVAAAEKTKQGVTEAAEKTKEGVMYVGTKTKEGVVQSVTSVAEKTKEQANVVGEAVVASVNTVANKTVEGAETIVATTGVVKKEDLAPQQPAAEGEAAIPGSTEGGGEGENEGN

>Anolis carolinensis_SNCG (ENSACAP_14476)

MDVFKKGFSIAKEGVVAAAEKTKQGVTEAAEKTKEGVMYVGAKTKEGVVQSVTSVAEKTKEQANLVGESVVASVNTVAKQTVEGAETVVSSTGVVKMEDLHPEQPEEPLADAEEDAPVEATETSPEGENEGY

>Xenopus tropicalis_SNCG (ENSXETP_7895)

MDVFKKGFSMAKEGVVAAAEKTKQGVTEAAEKTKEGVMYVGAKTKEGVVHSVSTVAEKTKEQANVVGGAVVSGVNQVASKTVEGTENIVGTTGLVKKEDLHPGQPEEPAAEEEPAVEATESTEQVGDGEN

>Danio rerio_SNCG (ENSDARP_123611)

MDVLMKGFSMAKEGVVAAAEKTKAGVEEAALKTKEGVMYVGNKTKEGMVSGVNTVVQKTTDQANIVGETAVGGANVAGQNTVEGLENVAASTGMVNPGDFSHGGMEGGEGGEGY

>Takifugu rubripes_SNCG (ENSORLP_13076)

MDVLKKGFSMAKDGVVAAAEKTKAGVEGAATKTKEGVIYVGNKTMEGVVTSVNTVAHKTTEQANIIADTAVSGANEVAQSAVEGVENAAVASGLVSLEEAGPVSEKAGVPNTEAEAEESEQVVQ

>Tetraodon nigroviridis_SNCG (ENSTNIP_4769)

MDALKKGFSMAKGGVVAAAEKTKAGVEEAATKTKEGVIYVGNKTMEGVVTGVNTVAHKTTEQANIIADTAVAGANEVAQSAVEGVENAAMASGLVKKEEAGPESEKAEAPGPEAEEEQSKQAAQ

>Gasterosteus aculeatus_SNCG (ENSGACP_3738)

MDVFMKGFSMAKEGVVAAAEKTKAGMEEAAAKTKEGVMYVGSKTKEGVVSSVNTVANRTVDQANIVGDTAVAGANEVSQAAVEGVENVAASTGMVNQGEYGGMEQGGEGGEGY

>Oryzias latipes_SNCG (ENSORLP_3349)

MDVLKKGFSMAKDGVVAAAEKTKAGVEEAATKTKEGVIYVGNKTMEGVVTGVNTVAQKSTEQVNVAAETAVAGANEVAEATVEGVENAAVASGFVSMEEAGPVAAEADLPKTAAGGEQNQPAAE
